# Optimization of Carrier-Based Dry Powder Inhaler Performance: A Review

**DOI:** 10.3390/pharmaceutics17010096

**Published:** 2025-01-13

**Authors:** Tanu Mehta, Saeed Najafian, Komalkumar Patel, Justin Lacombe, Bodhisattwa Chaudhuri

**Affiliations:** 1 Department of Pharmaceutical Sciences, University of Connecticut, Storrs, CT 06269, USA; tanu.mehta57@gmail.com (T.M.);; 2 Department of Chemical and Biomolecular Engineering, University of Connecticut, Storrs, CT 06269, USA; 3 Pharmaceutical Development & Engineering Department, Experic, Cranbury, NJ 08512, USA; 4 Institute of Material Sciences, University of Connecticut, Storrs, CT 06269, USA

**Keywords:** dry powder inhaler, carrier, formulation optimization, manufacturing, device design

## Abstract

Dry powder inhalers (DPI’s) are becoming increasingly popular due to growing interest in pulmonary drug delivery and their performance is the net result of a series of processes carried out during the formulation development and manufacturing process such as excipient selection, blending, milling, filling, and spray drying. To reach the small airways of the deep lung, the active pharmaceutical ingredients (API) particles need to have an aerodynamic diameter of 1–5 μm to avoid impaction and particle sedimentation in the upper respiratory tract, and due to this small particle size, the powder becomes highly cohesive resulting in poor flow. Therefore, API is usually blended with a coarse carrier to improve flowability, and due to its large size, it is more fluidizable than the micronized drug. Carrier-based DPI formulations usually consist of micronized drugs, a coarse carrier, and additional components, such as micronized lactose and force control agents, including magnesium stearate or leucine. Additionally, the manufacturing process of DPIs relies heavily on powder processing technologies, such as the micronization of API, blending, and powder filling. The aerosol performance of a DPI is significantly affected by the selection of formulation components and the processing of the formulation and, therefore, it is crucial to evaluate these parameters. This review will discuss different factors influencing the aerosol performance of carrier-based DPIs, including formulation components, device considerations, and manufacturing parameters. Additionally, novel technologies pertaining to the optimization of DPI performance are also discussed.

## 1. Introduction

The global pulmonary drug delivery market is expected to reach USD 41,072.7 Mn by the end of 2030 [1]. Pulmonary drug delivery has been recognized as an important route of drug delivery especially after the COVID-19 pandemic, as it offers multiple advantages such as minimized systemic side effects [2], targeted delivery to the lung, allowing lower doses than necessary [3], higher drug loading, and better formulation stability [4]. There are different types of devices for pulmonary drug delivery, of which dry powder inhalers (DPIs) and pressurized metered dose inhalers (pMDIs) are the most frequently utilized. pMDIs are one of the most commonly prescribed inhalers, as they are inexpensive to produce and develop. However, they are difficult to use due to their requirement for patient breath and actuation coordination to deliver the dose effectively [5,6]. In addition to that use of hydrofluorocarbon propellants in pMDIs has been known to cause roughly 0.03% of yearly global greenhouse gas emissions [7]. Therefore, making them detrimental to our environment. DPIs are gaining popularity for pulmonary drug delivery due to their propellant-free formulation and ease of use, as they are breath-actuated; therefore, no actuation inhalation coordination is required [2]. Aerohaler represents the first version of DPI, and it was used to administer penicillin powder to treat respiratory tract infections, followed by the approval of the SpinHaler DPI for the delivery of cromolyn sodium in 1971 [3]. Drug delivery using DPI is influenced by multiple factors, including the properties of the inhaler, inhalation rate, formulation components, and downstream processing of the formulation [8]. This article aims to provide a detailed overview of the factors influencing the dry powder inhaler performance and studies performed to evaluate these factors. Additionally, we will also present guidelines for designing a dry powder inhaler with optimum performance.

## 2. Formulation Parameters Affecting DPI Performance

**Figure 1:**
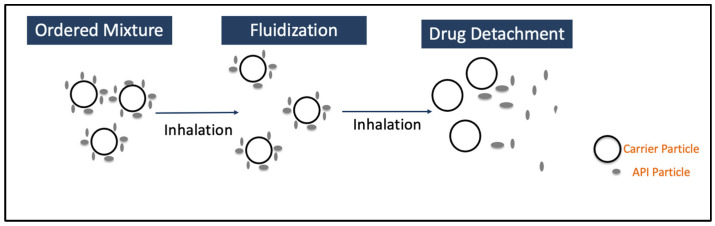
Working principle of DPIs.

The DPI formulation consists of micronized active pharmaceutical ingredients, along with other components or excipients, such as carrier (lactose or mannitol) [9], extrinsic fines (fine particle size sugars) [10], and force control agents (leucine and magnesium stearate) [11]. The key steps in delivering API to the lungs using DPIs include formulation fluidization, de-attachment of active drug particles from large carriers, and/or de-agglomeration of drug-only aggregates, dispersion, and transportation of the API particles, as well as deposition of these particles into deeper parts of the lung [12,13], as shown in Figure 1. Inhalation drug delivery is significantly influenced by the particle size of the API, particle shape, density, surface charge, hygroscopicity, breathing pattern (inhalation flow rate), device design, and excipient properties. In order to achieve effective delivery of API to deeper parts of the lung, the API particles should have a 1–5 mm aerodynamic diameter. The particles larger than 5 mm usually settle in the mouth–throat region [8]. Therefore, the API powder needs to be micronized to achieve particle size < 5 mm. The micronization of API generates highly cohesive Geldart group C particles that are difficult to disperse, as well as possess poor flow characteristics [14]. As group C particles are cohesive, the fluidization of these particles is challenging as the interparticle forces are higher than the forces imparted by the fluid or the airflow through the inhaler device [15,16]. These adhesive forces are a direct consequence of van der Waals forces, electrostatic forces, and capillary forces [14,17]. However, the overall contribution of van der Waals forces is still considered to be the most significant one. The fluidization of DPI particles could be explained by the drag and lift equation, as follows:

(1) $$F_{D,L}=C_{D,L}\frac{1}{2}\rho_{fluid}U^{2}\frac{\pi d_{g}^{2}}{4}$$

where $\frac{\pi d_{g}^{2}}{4}$ is the projected cross-sectional area of the particle, *U* is the velocity of the airstream, $C_{D,L}$ is either the drag or lift coefficient, and $\rho_{fluid}$ is the density of air [15]. As the API particles are cohesive and difficult to fluidize, they are often blended with coarse carrier particles usually lactose or mannitol to formulate blends also known as ordered mixtures [18]. The coarse carrier improves the flow properties, provides precise metering, and imparts higher momentum due to its size and density [19]. There are multiple studies performed to establish ways to determine the appropriate carrier for a particular formulation. While selecting a carrier, it is important to consider that it should be readily available, biocompatible, and safe to use in inhalation formulations, and upon inhalation, the API should easily detach from the carrier surface [20,21]. To achieve efficient detachment of API from the carrier surface, it is essential to optimize the cohesive–adhesive balance between the carrier–carrier and carrier–API particles.

The cohesive forces between the carrier particles and adhesive forces between the carrier and drug particle should be controlled to achieve the required detachment of the drug from the carrier surface, and high cohesive forces within the drug particles may lead to poor mixing and segregation of the mixture [22]. Therefore, it is extremely important to perform careful selection of carriers to achieve optimum aerosol performance. The performance of a DPI is significantly influenced by carrier properties, such as carrier type, carrier shape, size, and surface roughness. It is a common practice to evaluate different types of carriers to identify the best-performing carrier. Over the past few years, lactose has been the most commonly used carrier, and alternative sugars such as mannitol, glucose, dextrose, erythritol, glucose monohydrate, maltitol, maltose, mannitol, sorbitol, trehalose, and xylitol are also under evaluation [20,21,23]. It has been shown that high concentrations of mannitol can create a hypertonic environment and, therefore, are typically not preferred in DPI formulations for the management of asthma [24]. The aerosol performance can be evaluated using cascade impactors where the Next-Generation Impactor (NGI) is the most frequently used. NGIs represent a standardized and compendial in vitro technique designed for the evaluation of inhaled products. They classify particles based on their aerodynamic diameter, allowing for consistent measurement of their aerosolization and in vivo deposition behavior in a simplified manner [25]. The following sections discuss the modification of the above-mentioned formulation components to achieve optimum DPI performance.

### 2.1. Modification of Carrier Size and Surface Roughness

Numerous studies have been conducted to understand the impact of carrier size on DPI performance and to determine the optimum carrier size. It has been demonstrated by multiple research studies that decreasing the size of the carrier improves the aerosol performance but compromises the overall flow properties of the formulation, thereby making the powder handling difficult and leading to challenges associated with poor powder flow, such as content non-uniformity [17]. Steckel et al. (1997) evaluated the impact of carrier lactose size and API concentration on DPI performance [26]. In their study, two DPIs were investigated, single-unit-dose Spinhaler (Fisons) and multiple-unit-dose Easyhaler (Orion Pharma), to evaluate the deposition profile of lactose monohydrate and budesonide blend. The size of the lactose carrier was between 32 mm to 180 mm, where different size fractions were sieved and D50 of budesonide was kept below 3.1 mm. It was observed that as the carrier size increases with constant drug content, the fine particle fraction decreases [26]. In a study by Ooi, J. et al. (2011), three polystyrene sphere carriers with a D50 of 82.8 mm, 277.5 mm, and 582.9 mm and micronized salbutamol sulfate as the model drug [27] were presented. With respect to polystyrene carrier systems, data indicated that both carrier size and flow rate through the powder influence the drug deagglomeration through the device. Usually, the aerosol performance decreases with an increase in carrier size, as at the end of the inhalation, most of the drug particles remain attached to the carrier surface, and the number of carrier collisions also decreases. Moreover, higher drug liberation from the carrier surface was observed at high flow rates of 80–100 L/min due to increases in the energy input by the airflow on the drug–carrier liberation, which is explained in detail in later sections of this article. The energy input ($E_{dispersion}$) required to ensure sufficient dispersion can be calculated by using following equation [28]:

(2) $$E_{dispersion}=∆PV_{af}t_{dispersion}$$

where volume airflow is ($V_{af}$), a pressure drop across the inhaler is (∆P), and $t_{dispersion}$ is the time taken for powder to empty out of the capsule.

In a similar study by Du et al. (2014), granulated lactose with an average size ranging from 200 mm–1000 mm was evaluated as a carrier [29]. The granulated lactose was prepared using wet granulation and micronized salbutamol sulfate was used as the model drug. The coarse lactose granules (850–1000 mm) led to higher in vitro deposition under both 60 L/min and 90 L/min flow rates compared with smaller size lactose granules (600–850 mm), which could be attributed to the poor adhesion force between the drug and coarse lactose carrier. The carrier surface roughness, size, and bulk/tap density led to the observed decrease in adhesion force between the API and carrier particle. Lactose carrier particles could also be modified in a fashion to achieve higher aerosol performance. A coating of lactose carrier particles with lactose aqueous solution containing hydroxypropyl methylcellulose (HPMC) as a binder and micronized salbutamol sulfate was evaluated for the aerosol performance. The coated lactose carrier particles demonstrated significant improvement in the DPI performance when compared with uncoated lactose, which resulted due to decreases in surface roughness, thereby enabling easy liberation of drugs from the carrier surface [30]. Additionally, surface roughness is significantly influenced by particle size, and it has been shown that small particles usually have smoother surfaces, thereby leading to better drug detachment when API size is larger than the surface asperity [31]. However, one should always consider the impact of small particle size on powder flow properties. Surface roughness can be assessed using methods such as atomic force microscopy (AFM) or profilometry, which can offer detailed insights into the microscale surface topography [31]. Drug detachment is mostly influenced by the mechanical forces applied during the inhalation process, and weakly attached drugs are easier to detach than the API particles accumulated inside the large asperities of the carrier [32]. The next important question is to quantify how much surface corrugation is enough. In the study conducted by Chew et al. (2005), powders with varying degrees of corrugation (surface fractal dimension (Ds)) ranging from 2.06 to 2.41 [33,34] were prepared by spray drying, and FPF significantly increased from 27% to 41% when Ds varied from 2.06 to 2.18. However, Ds > 2.18 did not improve the FPF [35]. The increase in FPF was attributed to a reduction in capsule and device retention and an increase in powder dispersion [35]. It is important to note that the dependence of aerosol performance on carrier size is primarily controlled by the physical properties of the carrier. For example, the bigger spray-dried carrier particle demonstrated better performance than the small-size carrier particles [36]. Therefore, the carrier particle size must be optimized for a particular formulation to achieve optimum performance. In addition to that, an important property, known as Cohesive Adhesive Balance (CAB), should be carefully considered before selecting the carrier. CAB indicates the forces between API–carrier and carrier–carrier, and based on the previous studies, the carriers with a CAB ratio <1 have demonstrated higher FPF, which indicates it is better to have higher adhesion between drug and carrier particles than cohesion between drug particles [37,38].

### 2.2. Optimization of Carrier and API Morphology

The preparation of carrier-based DPI products begins by formulating an adhesive blend of carrier and API. The detachment of API particles from the surface of the carrier is controlled by multiple factors, in which API and carrier morphology play a huge role, as it controls the arrangement and attachment of the API particles on the carrier surface [39,40,41]. The carrier morphology can be characterized using elongation ratio, roundness, shape factor, and surface factor [42]. Elongation ratio (ER) is the ratio of particle length to particle width, and ER ≫ 1 indicates long and irregular particles [17,43]. Roundness (RO) indicates particle shape and surface roughness. Therefore, as roundness increases, the particle becomes more irregular due to an increase in surface irregularities, leading to increased surface roughness [44]. Particles with high ER and RO values experience more internal friction; therefore, they exhibit different aerosol performance [1,45]. For example, higher ER (approximately around 5) led to better aerosol performance [44,46,47]. However, the flow of the formulation becomes poor, which could lead to challenges during various unit operations such as blending and filling, eventually leading to poor aerosol performance. It is important to consider that the shape factor alone should not be relied upon for morphology assessments, as microscopy techniques are significantly influenced by the orientation of particles during the analysis [44]. In fact, particle morphology and particle size should be evaluated together, as they are dependent on each other [44]. Different drug particle morphologies could also be modified to achieve optimum performance. Adi et al. (2008) demonstrated that the slightly corrugated surface of bovine serum albumin (BSA) particles shows a higher fine particle fraction than smooth surface BSA particles from carrier lactose and BSA blends. The reduction in the contact area between the carrier and API surface leading to easy detachment was attributed to an increase in fine particle fraction [18]. In their study, the BSA particles were prepared by spray-drying to achieve varying degrees of surface roughness. The smooth particles were prepared at higher atomization pressure and higher feed rate, whereas lower feed rate and atomization pressures lead to particle collapse, producing rough particles [18]. Therefore, particle morphology, roughness, and contact geometry should be carefully evaluated [35].

### 2.3. Addition of Extra Components

DPI formulations are typically a complex mixture of components, such as carrier and API particles, and additionally, certain components such as fine lactose and force control agents. Usually, the excipients used are “Generally Recognized As Safe” (GRAS), specifically for the lung, and in comparison with other dosage forms, the number of excipients approved for inhalation formulations is very low [20,48]. Therefore, the optimization of formulation performance using excipients relies less on the choice of a type of excipient and more on the properties of the specific grade, which becomes really challenging for DPIs. However, if performed carefully, it could really help in improving aerosol performance. In the next sections, we discuss some of the potential components that can help in improving overall aerosol performance.

- Lactose fines: One of the most important factors controlling the release of drugs from the surface of carrier particles is the adhesive forces among the carrier and drug particles, and one of the ways to control these forces is by adding fine particle size components [49]. It has been observed in multiple studies that adding fines can increase the fine particle fraction [50]. The mechanism of this increase is related to fine lactose particles occupying the high-energy active sites present on the surface of the carrier and leaving the low-energy binding sites for the drug particles, including other mechanisms such as redistribution of the drug and change in dynamics of triboelectric forces [10,51]. The addition of inert fine carriers such as lactose particles is preferred over other fines due to the known safety profile of lactose. It is important to note that these high-energy active sites represent surface disorders resulting from dislocation in a crystal lattice or distortion of the crystal lattice, leading to exposed surface molecular groups and strong interaction with the drug particle. The drug particles have a higher affinity towards these higher-energy sites, leading to stronger interaction between carrier and drug particles. Therefore, it is a common practice to blend lactose fines and carrier particles before adding the drug. If the fines are added below the adhesive saturation limit of the carrier, the fines will preferentially occupy the high-energy active sites [19]. The other proposed mechanism for an increase in fine particle fraction is the formation of fine particle multiplets, which could either help in the dispersion of drug particles and ease of drug release from the surface of these multiplets, or these multiplets could be small enough to reach the deeper parts of the lungs [50]. Another important consideration is the concentration of added fines; it has been shown by previous studies that adding fines in the concentration of 10–15% leads to an increase in fine particle fraction including the concentration of fines already present in the carrier [10]. This increase in FPF was on the order of 60–70%. However, adding fines in very high concentrations often leads to a decrease in FPF, which could be attributed to an increase in the number of bigger-sized fine aggregates [10]. Also, added fines may compromise the flow of the formulation making the powder handling difficult for processes such as blending and encapsulation [52]. The selection of fine concentration varies for different types of carriers due to the variation in surface characteristics of different carriers [10]. The theoretical surface area calculation could provide an initial estimate of the highest fine load. It has been shown in previous studies that the chemical nature of fines plays a less significant role in improving the respirable fraction of the formulation [23]. It is also important to consider the amount of intrinsic fines in the carrier as they also contribute to the improvement in aerosol performance. It was demonstrated by Islam et al. that binary blends of salmeterol xinafoate and various grades of carrier lactose when mixed with increasing concentrations of fine lactose led to an increase in the FPF. However, the increase in FPF was only observed up to 15%, and a further increase in fine concentration did not produce a change in FPF. Additionally, the concentration of fines can also be controlled by removing them externally using methods such as air jet sieving, air washing, or decantation. However, one should also consider the impact of change in surface characteristics imparted by the method of fine removal. For example, air jet sieving and air washing of fines might introduce electrostatic charging, which could lead to a decrease in the formulation aerosol performance [53].
- Force control agents: In addition to adding sugar fines, force control agents (FCAs) are also added, such as leucine, lecithin, and magnesium stearate [11], to improve the aerosol performance of DPIs [54]. Magnesium stearate (MgSt) is one of the most used FCAs and has also been employed in marketed products, such as Novartis’ seebri Breezhaler, Ultibrom Breezhaler, and GSK’s Relvar Ellipta, Anoro Ellipta, and Incruse Ellipta [55]. FCAs are known to improve the aerosol performance of a DPI formulation by occupying high energy active sites on the surface of the carrier particles in a similar fashion to extrinsic fines thus creating a surface smoothening effect leading to ease of drug detachment from the surface of the carrier particle [54,56]. Additionally, the method of blending carriers with FCA impacts the overall aerosol performance. For example, high-shear blending has been shown to produce blends with better aerosol performance [57]. There are multiple mechanisms explaining the increase in aerosol performance using FCAs. One of the mechanisms is related to the ease of removal of drug particles from magnesium stearate carrier particles, which could be attributed to the weaker interaction of the drug with the carrier, as demonstrated by previous studies. This interaction could also be measured by the work of adhesion between the API–carrier and the work of cohesion between API-API particles, as shown in previous studies [58]. It was established that the work of cohesion between SS particles was 407.5 mJ/m^2^, and the work of adhesion between SS and MgSt was 311.6 mJ/m^2^. Therefore, it was easier to detach SS from the surface of the carrier [58]. It was also established by Li et al. (2023) that 0.25–0.5% is the optimum concentration using both low- and high-shear blending for optimum aerosol performance. They also established that powder rheology evaluation can also be useful in predicting factors that affect aerosol performance [55]. Additionally, it is also important to consider the concentration of MgSt that is allowed by the FDA in per unit dose is 0.13 mg as per the inactive ingredient database [59,60]. The distribution of FCAs on the surface of carrier particles can be evaluated using multiple methods, such as Atomic Force Microscopy, Raman spectroscopy, or Time-of-flight secondary ion mass spectrometry (ToF-SIMS) and X-ray photoelectron spectroscopy (XPS) [61]. The following Table 1 lists some of the most commonly used DPI excipients, their functionality, and their development stage.

### 2.4. Optimization of Drug Loading

The influence of drug loading on DPI performance has been evaluated by many researchers [9,66]. The drug loading significantly influences the drug deagglomeration from the carrier surface, and mostly, a decrease in aerosol performance is observed. However, this phenomenon is observed up to a certain API concentration, and additional API loading results in improved aerosol performance [66]. The improved aerosol performance could be attributed to the preferential occupation of high-energy active sites by some of the drug particles and the remaining drug particles occupying the comparatively low-energy sites [66]. The drug concentration for carrier-based DPI formulations should be increased up until the point of active carrier site saturation and, after that, the aerosol performance significantly deteriorates due to the formation of drug particle agglomerates that are difficult to disperse [66]. It is crucial to consider the size of the lactose carrier while determining the appropriate API loading. Large-size carriers should be used when high API-loaded formulations are prepared; however, smaller drug loading should be performed while using a small carrier size [67]. The appropriate drug and fine content could be determined by considering methods such as optical microscopy, sieve analysis, and powder bed permeability, as described by Benassi et al. in 2019 [68]. During sieve analysis, the non-bonded fines are sieved with minimal vibration of 50 Hz and oscillation amplitude of 0.2 mm to avoid detachment of fines from the carrier surface. The amount of fines collected gives an estimate regarding the surface saturation of the carrier. The permeability assessment of the blend could be really useful in understanding the presence of fines on the carrier surface or the interstitial spaces between the carrier particles [69,70]. Lastly, analysis of surface coverage through optical imaging by observing the regions with fine particles and determining the coverage index thereafter [68]. The coverage index is defined as the ratio between the area occupied by the fines over the total particle area, and it generally ranges between 0 and 1.

### 2.5. Impact of Capsule Type

In addition to the formulation components, the type of capsule is known to affect the aerosol performance of DPI. The assessment of capsule properties, including moisture diffusion, permeability, physical and mechanical performance under puncturing or cutting conditions, and the presence of lubricants on the inner surface, is crucial [71]. The most used capsules for DPIs are made of gelatin, gelatin–polyethylene glycol, hydroxypropyl methylcellulose (HPMC). The DPI capsule has direct contact with the formulation. Therefore, it is important to study the impact of capsule type on the DPI performance, and capsule type should be evaluated to optimize the DPI performance [72]. Generally, HPMC capsules are preferred over gelatin capsules, as they contain significantly less water (approximately 4–6%), minimizing the risk of brittleness during storage under low relative humidity conditions. In a study by Wauthoz et al. (2018), the impact of gelatin capsules (Qualicaps QualiG™), cold-gelled HPMC capsules (Vcaps plus™), and thermal-gelled HPMC capsules on delivered dose (DD), fine particle dose (FPD), and capsule retention for formoterol–lactose binary and ternary blends using a low resistance RS01 device was studied. Higher DD and FPD were observed with cold-gelled HPMC capsules, which could be attributed to the lower drug retention in the capsules in comparison with other capsule types [73]. It was also demonstrated that capsule type significantly affects the aerosol performance especially when carrier-free rifampicin formulations are used [72]. However, no significant difference in aerosol performance was observed for carrier formulations when Embocaps and ACGcaps were compared. Embocaps in general showed consistent performance when compared with ACGcaps for carrier-free formulations, and it was attributed to the hardness of the capsules. It is known that capsule hardness affects the powder dispersion by controlling the collision velocity between the capsule and the inhaler wall and an increase in capsule hardness increases the impaction forces facilitating the powder dispersion [74]. A comparative study also indicated that the aerosol performance of micronized ciprofloxacin powder filled in gelatin and HPMC capsules is notably affected by the size and geometry of the punctured capsule hole. Gelatin capsules led to higher drug content being delivered through larger, irregularly shaped holes, leading to elevated emitted dose (ED) values compared with HPMC. However, the deagglomeration efficiency of particles was compromised, resulting in decreased FPF [75]. Moreover, it is important to consider the capsule size, for example, a larger number of collisions were observed with larger capsules but also led to increased powder retention in the inhalation device after use. Therefore, it is crucial to balance this with the lower drug loading of the smaller capsule, potentially necessitating an increased number of doses to be loaded and inhaled by the patient to achieve a therapeutic effect [76].

## 3. Inhaler Device Consideration

DPI performance is also significantly affected by the type of inhaler device and design. There are multiple devices, approved and under evaluation for delivery of carrier-based inhalation formulations [77]. There are different types of inhaler devices based on their mechanism for powder dispersion: active inhaler devices where the powder dispersion is facilitated by the powered fan and passive inhaler devices where the dispersion is controlled by the patient’s inhalation pattern and health condition. In summary, it is important to consider the effects of device flow rate along with the formulation components.

### 3.1. Flow Through the Inhaler Device and Device Resistance Optimization

The flow through the device should be designed and controlled in a way to ensure optimum deagglomeration of the carrier–drug agglomerates. Also, minimizing the amount of drug retained inside the device and the capsule. The deagglomeration is controlled by the forces exerted by the air on the particles, resulting in events such as particle–particle, particle–wall collisions, vibrations, and centrifugal acceleration [28,78]. The energy required to initiate the powder deagglomeration could be estimated by continuity and Bernoulli’s equation, as described by Equation (2). Additional energy could be required for devices with capsule spinning [28]. It has been shown that higher dispersion is observed when the capsule rattling is high leading to higher deagglomeration due to particle—particle and particle–wall collisions. The impact of flow through the inhaler device on the powder deagglomeration can be studied experimentally; however, it is difficult to completely understand the underlying mechanics and physics using experiments. Therefore, computational, or numerical modeling could be a useful tool in studying and understanding the underlying mechanics of deagglomeration and the impact of different factors, such as device, capsule, formulation properties, and inhalation conditions on overall inhaler performance. This improved understanding will eventually be useful in optimizing parameters such as flow through the device, capsule piercing, deagglomeration, and device emptying for optimum product performance. Initial simulations could also decrease the number of experiments required to develop the device [79,80,81]. Multiple studies have employed numerical modeling to study the impact of various factors on DPI performance. The fundamentals regarding modeling and simulation are beyond the scope of this article and can be found elsewhere [76,82,83,84,85]. Computational fluid dynamics (CFD) analysis was performed to understand the flow field generated in the Aerolizer when the mouthpiece and inhaler grid were modified [81]. It was observed that the inhaler grid significantly influences DPI performance, and an increase in grid voidage generally decreases DPI performance, mostly due to increased retention of powder in the device. Flow through the device provides the required force for fluidization and deagglomeration, which is controlled by the inhaler device resistance and patients’ inhalation effort [86,87]. A recent study by Ye et al. (2022) investigated the effect of 3D0-printed DPI grids on aerosol performance by employing experimentally validated CFD modeling, and they established that intensive grid meshes with a small void size improve aerosol performance by increasing the turbulent kinetic energy [88]. In addition to CFD analysis, it is important to consider the presence of particles (API and carrier) in the formulation, where a CFD-DEM (Discrete Element Method) coupled model could also be employed [88]. DEM is commonly used for simulation particles in a system [89]. It has been established by multiple studies that in addition to factors such as device design, flow rate through the device, and pressure drop achieved, the patient’s inhalation effort during the inhalation process highly influences the deagglomeration of the formulation, which is eventually controlled by the device resistance. For example, high-resistance devices usually lead to a lower flow rate, with higher turbulence generated in the device and more deagglomeration, but a higher flow rate might not be achievable due to the patient’s condition. Therefore, it might be tempting to utilize high-resistance devices, but they might not prove that effective, and device selection should always be performed after careful consideration of the patient’s condition, as well as formulation properties.

### 3.2. Optimization of Device Design

One of the first DPI manufactured on a large scale, Aerohaler by Abbott, was fabricated by incorporating small capsules of a lactose-based formulation, and shortly after that, multidose inhalers started gaining popularity. Although they had similar formulations, their dose-measuring mechanisms were different, including slides (e.g., Novolizer^®^, Meda Pharma), cylinders (e.g., Easyhaler^®^, Orion Pharma), and disks (e.g., Pulvinal^®^, Chiesi Farmaceutici). DPIs with carrier-based formulations were introduced, such as Jethaler^®^ (Ratiopharm) and MAGhaler^®^ (Mundipharma), where the formulation was compressed into a tablet with a circular shape which gets deagglomerated during the inhalation process. MAGhaler is a device that consists of a mechanically wound drive unit designed for extended usage, integrating a grinding wheel equipped with a face-cutting mechanism. Upon the patient’s activation of the device by pressing a button, a precisely measured amount of medication (such as salbutamol sulfate) is milled from the frontal surface of the ring-shaped tablet. Subsequently, the patient inhales the drug powder. This system allows for consistently accurate dosing, and it also protects the powder from moisture until the dose is released [90]. NEXThaler DPI is a multidose device where the drug can be inhaled only with an airflow of 35 L/min, which is called a breath-actuated mechanism (BAM) that facilitates deagglomeration with minimal effort by the patient. Similarly, there are multiple products in the market that have been successfully used to improve the aerosol performance of the formulation. One such example is the use of cyclones in devices like Cyclohaler, where substantial tangential velocity and turbulent energy introduced by the cyclones can help with the separation of API from the carrier particle [48]. It becomes really challenging to produce adhesive mixtures that can withstand handling and have good flowability but are also weak enough so that API can be detached from the carrier surface. Therefore, mini cyclones could be useful in ensuring efficient API detachment. Additionally, reducing the diameter of the outlet and decreasing the cone height results in an elevation of the tangential velocity, higher static pressure near the cyclone wall, and lower static pressure at the center of the cyclone, leading to easy detachment of API from the carrier surface [91]. Infant air jet DPI is also a new and innovative approach to improve the efficient and high-dose drug delivery of Albuterol sulfate. This DPI platform consists of three essential components, the air source, air jet DPI, and the nasal interface. The air source provides the energy required for aerosolization of the formulation which makes the inhalation process easier for the infant. Additionally, critical components such as the entry pathways for air, the chamber where aerosol is produced, and the capillary that transfers the aerosol to the nasal interface control the efficiency of formulation delivery [92]. In a recent study published by Chaugule et al. (2023), a novel design concept was evaluated that can help in passively optimizing the DPI performance. In this design, a varying inner cross-section of the pipe-shaped device realized in the form of converging and diverging circular sections was evaluated, where 68.4% FPF was achieved, in comparison with 66.8% from the baseline device [93].

## 4. Process Parameters

The impact of process parameters on DPI performance has been shown to be significantly affected by the processes of powder handling during the manufacturing process, such as blending, milling, powder filling, or encapsulation [94]. The following section lists factors and studies conducted to evaluate different process conditions.

### 4.1. Blending

In order to improve the flowability of the formulation, API is usually blended with carrier particles to produce an ordered mixture [95]. Therefore, it is important to optimize the blending process. Different types of blenders could be used, including high shear or low shear, which are selected according to the type of formulation to be processed. There are multiple studies that have been performed to evaluate the influence of blending conditions on DPI performance [96,97,98,99]. The effectiveness of low-shear was compared with high-shear blending, and it was observed that high-shear blends produced high FPF, which was attributed to an increase in the amount of fines, thereby changing the powder fluidization characteristics [100]. In Hertel et al. (2017), the influence of high-shear mixing process parameters on DPI performance was studied. A blend consisting of coarse lactose (Inhalac 70 and Inhalac 230), lactose fines (Inhalac 400), and micronized budesonide was prepared and evaluated. It was established that longer mixing times and high rotation speeds can lead to lower FPF [94]. The decrease in FPF could be attributed to the increase in the press-on forces of API onto the carrier, leading to difficulty in API detachment from the carrier surface. The impact of rotation speed on press-on forces could be estimated by calculating the energy consumption during the mixing process where high energy consumption indicated higher press-on forces [101]. Additionally, higher speeds (~500 rpm) also led to the milling of the carrier particles during the mixing process, which could increase the concentration of fines. However, this was not observed for the mixtures, as more drug particles were pressed onto the blender walls due to high shear [94]. Therefore, in general, the mixtures should be mixed for short durations to minimize high press-on forces. Recently, Spahn et al. (2022) evaluated twin screw extruders for continuous mixing of adhesive DPI mixtures to produce a formulation consisting of micronized rifampicin (1%), magnesium stearate (0.4%), and lactose carrier. The blending efficiency of the twin screw extruder was compared with the low-shear Turbula mixer, and it was established that the twin screw extruder produced desirable blend uniformity and aerosol performance, along with its ability to support continuous manufacturing [63]. The FPF observed with formulation processed using a twin screw extruder, and Turbula blender was found to be ~28% and ~20%, respectively, which could be attributed to improved mixing using a twin screw extruder. The improved mixing results in improved distribution of magnesium stearate, leading to increased pacification of high-energy sites [63]. Therefore, an appropriate selection of blending methods could be really useful in improving the overall product performance, and blending process optimization should always be performed to improve the aerosol performance.

### 4.2. Milling

To produce an effective DPI formulation, the size of API and carrier particles should be strictly controlled. There are different types of milling designs that could be used for the micronization of the material, such as fluid impact mills, jet mills, spiral jet mills, and oval chamber mills [102,103]. Jet milling is one of the most commonly used mills for DPI manufacturing; however, the end product from jet milling is usually highly cohesive material and prone to tribocharging [104], although tribocharging can be handled by implementing various strategies, as described elsewhere [105,106]. Another commonly used approach is ball milling, where grinding media (balls) are used. Ball mills can handle fairly small (~1 g) amounts of material. In a study by Steckel et al. (2006), the influence of milling conditions, such as milling intensity, was evaluated on the flow properties and physicochemical characteristics of lactose crystals. It was observed that gentle milling results in morphological surface defects, which further increase with the increase in milling intensity, resulting in improved FPF. Therefore, it is essential to evaluate the impact of milling conditions on aerosol performance in addition to achieving the desired particle size [107]. It might be useful to pretreat the carrier particles by milling to improve aerosol performance. Additionally, the influence of the type of milling process should also be taken into consideration. The modified pearl mill, when used for the production of GnRH-antagonist cetrorelix, demonstrated better aerodynamic performance when compared with the spray-dried formulation, which highlights the importance of the milling process in improving formulation performance [108]. Co-jet milling of the API with lubricants can also be useful to improve the aerosol performance of a formulation. Mangal et al. (2019) evaluated co-jet-milled ciprofloxacin HCl with lubricants such as magnesium stearate (MgSt) or leucine to improve aerosolization performance [109]. Co-milling facilitates the micronization process in addition to improving the overall content uniformity of the formulation [110]. Additionally, wet milling has also been shown to significantly improve aerosolization and solubility of magnesium stearate and leucine-embedded inhalable Ibuprofen formulation [62]. The micronized product is highly prone to recrystallization due to the creation of amorphous regions during the micronization process [111]. The recrystallization results in uncontrolled particle growth, compromising aerosol performance. However, the preconditioning of micronized salbutamol sulfate (SS) at 55% RH at room temperature is suitable to ensure minimum particle growth [112]. Hence, preconditioning can be investigated as a method to minimize particle growth following micronization.

### 4.3. Filling/Encapsulation

DPIs are usually marketed as unit-dose or multidose, and usually, the formulations are in the order of milligrams, which becomes really challenging to achieve during the manufacturing process. There are multiple methods that exist for DPI powder filling, such as volumetric filling (dosator, vacuum dosator, vacuum drum filling, tamp filler) and gravimetric filling (microdosing using dosator systems, vacuum drum filling, membrane filling) [112,113,114,115,116]. Usually, different formulation components are evaluated and modified to produce DPIs with optimum performance. However, it is important to consider the impact of the manufacturing process on the overall formulation performance. The standard dosator technology was first introduced in the 1970s, where dosator heads plunge into the powder and are pulled back, and a dosing plug is collected into the dosator and ejected into the capsule or a receptacle [117,118,119]. Usually, the slug compression forces dissipate as the piston is retracted, but in some cases, a retention force could be observed [120]. The amount of powder dispensed is determined by the level of the dosing piston. The amount of powder can be filled in the range of 10 to 500 mg with a very low %RSD. One of the studies investigated two carriers (lactose and mannitol) blended with spray-dried salbutamol sulfate blended and filled into capsules using a dosator machine at different process conditions. The filled capsules were also evaluated for their aerodynamic performance. The DPI formulation manufactured using lactose and a dosing chamber-to-powder layer (compression) ratio of 1:2 performed significantly better than other conditions with an FPF of 12% and filling %RSD of <0.8% [121].

**Figure 2:**
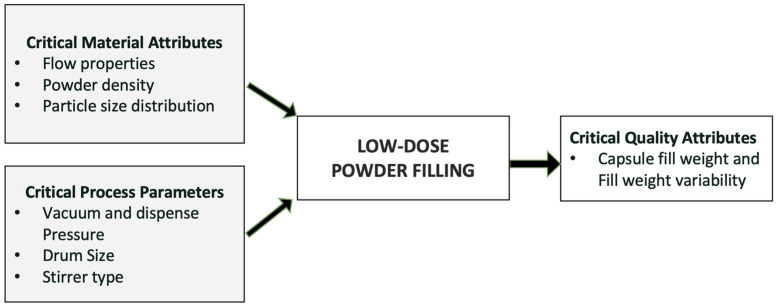
Critical process parameters and critical material attributes during low-dose powder filling.

Vacuum drum filler is also one of the most commonly used types of powder filler, as it allows minimum compression of the powder and leads to the lowest effect on the powder properties [116]. There are multiple pieces of equipment available in the market utilizing vacuum drum filling systems. A vacuum drum filling system is highly useful for filling cohesive and low-dose formulations with much less fill weight variability. Some of the most commonly used examples of this technology are Harro Hofliger DrumLab, ModuC LS, and Omnidose. A vacuum drum filler involves a rotating drum with bores of different sizes [113,122]. The powder is pulled into the drum bore by vacuum and dispensed into the capsule or receptacle with positive pressure. The amount of powder dispensed is controlled by multiple factors, such as vacuum pressure, drum bore size, type of stirrer, stirring speed, and powder properties, as shown in Figure 2. Sibum et al. (2020) investigated the influence of filling conditions on dispersibility using Omnidose for high-dose Amikacin formulation. Observations revealed that altering the filling conditions did not result in a significant difference in fine particle fraction (FPF). Moreover, as the packing fraction of the dosing pellets increased, the nominal dose also increased [122]. Stranzinger et al. (2023) evaluated a novel microdosing system to fill powders in low doses ranging from 0.5 mg to 10 mg. The system comprised a product hopper, a stirrer, an auger, a dosing chute, and in-process weight control [123]. Using the proposed dosing system, cohesive materials (Lactohale 300, Lactohale 220, and Inhalac 500) were filled with average fill weights of 0.48 mg (%RSD < 11%), 1.01 mg (%RSD < 7%), and 10.00 mg (%RSD < 2.30%). The utilization of the innovative powder dosing system confirmed its capability to achieve successful dosing with an acceptable to good level of dosing accuracy. This is particularly crucial when dealing with low doses of formulations and a narrow therapeutic window [123,124]. Therefore, it becomes essential to optimize the filling conditions, as they eventually impact the DPI performance.

Moreover, the Design of Experiments (DOEs) could also be implemented early in the development process to identify critical process parameters. DOEs is a common systematic approach to evaluating the effects of multiple factors on the performance of DPIs, making it a critical tool for identifying and optimizing parameters such as particle size, excipient concentration, device design, and airflow properties [125]. By investigating these factors in relation to specific APIs, DOEs enables researchers to understand complex interactions and optimize formulations for efficient drug delivery. PSD is a particularly critical parameter for DPIs, as the optimal size range (1–5 µm) ensures efficient deposition in the respiratory tract. DOE studies can evaluate how manufacturing conditions, such as milling time and speed, affect PSD and aerosolization efficiency, allowing for tailored optimization of API properties. Formulation composition, including the type and concentration of excipients like lactose or mannitol, also significantly influences DPI performance by affecting flow properties and dispersibility. Factorial designs within DOEs help in the identification of optimal excipient concentration and their impact on critical performance metrics such as fine particle fraction (FPF) and emitted dose [126]. For instance, device design plays an equally vital role, as parameters such as airflow resistance and mouthpiece geometry directly impact powder dispersion and consistency. DOE approaches can assess how these factors interact with formulation characteristics to achieve reproducible performance. Biologics, which present unique stability challenges, benefit from DOEs in optimizing stabilizer concentrations, excipient selection, and spray-drying conditions to preserve biological activity while enhancing deposition profiles. Real-world applications of DOEs underscore its versatility. For example, studies based on DOEs utilized a Quality by Design (QbD) approach to optimize the development of budesonide (BUD)-loaded large porous microparticles (LPPs) for inhalation therapy. Critical factors were identified using an Ishikawa diagram, followed by single-factor studies to assess the impact of drug loading and polymer type on LPP properties. A central composite design was then employed to evaluate the influence of high-risk parameters, including O/W phase ratio and polymer concentrations (PVP and PVA), on key quality attributes, such as particle size, fine particle fraction (FPF), and encapsulation efficiency (EE). Optimized LPPs were achieved with >60% EE and ~6 μm aerodynamic particle size (FPF > 21%), demonstrating the effectiveness of QbD in developing inhalable formulations.

Similarly, investigations into device design for high-dose DPIs have shown that medium airflow resistance and optimized mouthpiece geometry improve drug delivery efficiency. In biologics, DOEs has been used to identify stabilizer concentrations and spray-drying parameters that maintain API stability while ensuring efficient lung deposition. Overall, DOEs provides a robust framework for systematically identifying critical parameters, optimizing formulations and devices, and ensuring reproducibility across batches, ultimately enhancing the effectiveness and reliability of DPI therapies for a wide range of APIs [125].

## 5. Impact of Particle Engineering

Particle engineering plays a critical role in optimizing the performance of dry powder inhaler (DPI) formulations. By precisely controlling particle size, shape, and surface properties, particle engineering ensures that drug particles exhibit improved aerodynamic behavior, leading to their optimal deposition in the lungs upon inhalation. Moreover, particle engineering enables the development of stable DPI formulations with improved flow properties, ensuring consistent dosing accuracy and patient compliance. In essence, particle engineering serves as the cornerstone of DPI technology, driving innovation and advancements in pulmonary drug delivery for enhanced therapeutic outcomes. Various approaches, including interactive powder blends and engineered particles, supported by techniques like spray drying, exemplify the breadth of applications and innovations in DPI formulations. Particle engineering involves techniques like micronization, spray drying, and supercritical fluid processing, enabling the production of particles within the respirable range (<5 µm) with an overall goal of optimizing interparticle interactions, surface energetics, and particle dispersion [49], thereby enhancing the inhalation efficiency and lung deposition. Particle engineering also involves surface modification strategies, such as the addition of force control agents (FCAs), optimization of surface roughness, and surface coating of carrier particles [49].

### Particle Size Control Techniques

- Micronization or Milling: As discussed in previous sections of this article, there are multiple techniques for milling APIs, with air jet and ball mill being the most commonly used ones for DPI manufacturing. An air jet mill consists of a cylindrical grinding chamber equipped with tangential nozzles for directing the air. High-velocity gas jets accelerate the particles, leading to collisions between particles, as well as between particles and the chamber walls. Within the mill chamber, particles experience both centrifugal and drag forces. The centrifugal force pushes the particles toward the chamber’s outer edge, while the gas’s radial velocity component creates a drag force that pulls the particles toward the central outlet. Particles exit the chamber when the radial drag force surpasses the centrifugal force. The key parameters to be optimized include the feed rate, feed pressure, and grind pressure [127,128]. The operation of a ball mill involves both crushing and grinding actions, primarily carried out by large and small balls, respectively. Achieving a balanced combination of crushing and grinding requires an appropriate distribution of ball sizes. In many industries, it is standard practice to use a mixture of ball sizes rather than balls of uniform size. This approach ensures efficient grinding of API particles of varying sizes [129]. In one study using salbutamol sulfate, the grind pressure played a crucial role in determining the particle size distribution and ensuring the physical stability of the powder and feed rate had minimal impact on the quality of the powder. A higher feed rate led to a greater concentration of product in the grinding chamber and reduced the distance between particles, resulting in coarser particles. The feed pressure did not significantly affect the particle size [127]. Another study evaluating inhalable microparticles of Amifostine (AMF) using wet ball milling and jet milling demonstrated that microparticles wet-ball-milled with non-polar solvents exhibited higher inhalation efficiency compared with wet-ball-milled with polar solvents. Additionally, wet ball milling outperformed jet milling across all samples. Wet ball milling using a polar solvent led to a decrease in hydrate content and led to a modification of crystallinity and inhalation efficiency. In wet ball milling, the crystal structure is influenced by changes in hydrate content influenced by the solvent polarity. Moreover, the transformation of the unstable form (dehydrated) into a stable form (trihydrate) reduces agglomeration due to the hygroscopic nature of free water, thereby decreasing inhalation efficiency. Hence, non-polar solvent wet ball milling, which better maintains stable crystallinity and high inhalation efficiency, is preferable for producing inhalable particle sizes [130] In another study, the difference between wet and dry ball milling was discussed by employing quartz as the model API. The final powder product size, specifically the median particle size at the grinding limit, was approximately 2 µm under dry conditions and around 0.5–0.6 µm under wet conditions. Thus, the particle size achieved through wet grinding was about one-fourth of that achieved through dry grinding. Also, particle size distribution by ball milling achieved through wet grinding with small balls was narrower. Conversely, the broader particle size distribution was achieved during dry grinding with small balls [131]. However, it is important to take into consideration the influence of ball milling on the crystallinity of the API. In general, ball (wet and dry) milling introduces high mechanical stress, causing surface defects and leading to amorphization. However, milling performed above Tg can induce crystallization due to increased molecular mobility. Therefore, it is important to evaluate different milling techniques in addition to optimizing final particle size and product performance.
- Spray-Drying: Spray-drying has been gaining popularity in DPI manufacturing due to its ability to efficiently create respirable particles. Particles created via spray-drying generally have a spherical shape, with aerodynamic diameter usually between 1 and 5 µm and exhibiting a narrow size distribution. Additionally, it offers the advantage of generating carrier-free DPI formulations [64]. Typically, spray-drying involves multiple critical steps, where first one is the atomization of feed solution into a spray, drying of the atomized droplets at high temperatures, and separation of dried particles from the air followed by collection of these particles [49,132]. Several elements affect the properties and aerodynamic behavior of these powders, such as the design of the nozzle, the viscosity of the solution being fed into the system, and the temperature at the outlet [133]. Additionally, the carrier selection also influences the spray-drying and consequently DPI performance. For example, when azithromycin was evaluated with different carriers (lactose, mannitol, L-leucine, and glycine), the scanning electron microscopy images of powders containing L-leucine exhibited the most uniform distribution. Using lactose as the carrier caused the particles to become wrinkled, while mannitol produced particles that were more spherical and smoother. However, the respirable fractions values were observed in decreasing order of L-leucine > glycine > lactose > mannitol. Mannitol exhibited the lowest in vitro deposition of the drug because it produced the largest particle size with broad PSD. The lower RF of lactose may be attributed to its poor flowability. The highest RF achieved with a formulation containing leucine was attributed to two main factors: good flowability and suitable particle size with a narrow PSD [134]. In another study, the effect of solvent on DPI formulation was investigated, where ethanol was evaluated as the solvent and β-estradiol as the model drug. Raising the ethanol concentration in the solvent before spray-drying reduced the yield of the resulting powder. Conversely, the powder yield improved with increasing concentration of leucine in the formulation [135]. In a different study, jet milling and spray-drying were compared using bosentan hydrate as the drug. The spray-dried particle exhibited a spherical and smooth morphology with an amorphous solid state. This could be due to the rapid evaporation of the solvent during spray drying, which allows insufficient time for crystallization. On the other hand, the jet-milled samples exhibited an irregular shape with a rough texture and a crystalline solid state with relatively high moisture content. The irregular and non-spherical form of jet-milled samples contributed to a smaller aerodynamic diameter, resulting in better aerosol dispersion [136].

Additionally, the development of amphiphilic excipients and core–shell particle designs has significantly enhanced the ability to produce microparticles with refined aerodynamic properties and improved stability, crucial for effective pulmonary drug delivery. One of the major advancements in this area is the creation of lactose-free microparticles, which offer an alternative for individuals with lactose intolerance while also enhancing compatibility between the drug and excipients. For instance, mannitol-based formulations prepared through spray-drying have demonstrated superior stability and aerosolization compared with conventional lactose-based systems [137]. Another noteworthy innovation involves the integration of active carriers such as heparin and mannose into spray-dried formulations. Heparin–azithromycin microparticles have shown dual functionality by providing anti-inflammatory benefits and combating bacterial pathogens, including those associated with SARS-CoV-2 infections [138]. Likewise, mannose-functionalized particles have been designed to interact with specific lung cell receptors, enabling precise therapeutic targeting for conditions like fungal and parasitic pulmonary infections [139]. In conclusion, these innovations highlight the transformative role of spray-drying in creating customized, high-performance inhalation therapies aimed at enhancing drug delivery to the lungs.

- Supercritical Fluid Processing (SCF): Supercritical fluid processing is a sophisticated technique utilized in DPI formulations to achieve precise control over particle size and morphology. It involves the use of supercritical fluids, such as carbon dioxide or ethane, as solvents or antisolvents in particle formation processes [140]. By adjusting pressure and temperature conditions, supercritical fluid processing enables the production of particles with tailored properties, including size, shape, and surface morphology. This technique offers several advantages over conventional methods, including the absence of organic solvents, low processing temperatures, and high product purity. Supercritical fluid processing is particularly suitable for producing nanoparticles and microparticles with narrow particle size distributions (PSD) and uniform properties, making it a promising tool for enhancing the performance of DPI formulations. However, this might not be applicable to all API’s. For example, Richardson et al. (2007) evaluated salbutamol-sulfate-containing drug substances manufactured by supercritical fluid as compared with marketed micronized formulation, and no significant differences were observed in inhalation performance. Moreover, the DPI performance for Salbutamol manufactured using SCF processing led to more inter-batch variability in inhalation performance [141]. Additionally, Rehman et al. (2004) evaluated Terbutaline Sulfate (TBS), and it was observed that SCF led to better aerosol performance, with particle sizes consistently within the respirable range [142].
- Electrohydrodynamic Atomization: Electrohydrodynamic atomization (EHDA) is a technique used in the development of DPI formulations to produce fine and uniform particles through electrostatic forces. EHDA involves the generation of a high-voltage electric field between a liquid formulation and a grounded electrode, inducing the formation of a Taylor cone at the liquid–air interface. As the electric field strength increases, the liquid jet emanating from the Taylor cone undergoes rapid stretching and fragmentation, resulting in the production of fine droplets. These droplets subsequently evaporate to form dry powder particles with controlled size and morphology. The drop size (D) or consequently the particle size is controlled by the liquid flow rate and the fluid properties, such as conductivity, surface tension, and density, and it can be predicted by the following Equation (3):

(3) $$D=0.767c\;{\left(\frac{\rho \varepsilon }{\gamma K}\right)}^{1/6}\;Q^{1/2}$$

where *c* is a constant, ρ is the density of the liquid, ε is the permittivity of the free space, K is the conductivity of the liquid, Q is the applied flow rate [143]. Ijsebaert et al. (2001) evaluated EHDA for the development of a DPI for beclomethasone dipropionate at different process conditions. It was observed that higher flow rates resulted in an increase in particle size (1.58 µm at 1 mL/h vs. 4.55 µm at 3 mL/h) [143]. Additionally, no significant variations within particles were observed at similar process conditions. This technique could be employed for heat-sensitive drugs, as it operates at ambient temperatures and does not require the use of organic solvents. It is important to note that this process requires the use of a volatile solvent most commonly ethanol, and therefore, solubility becomes a significant concern and should be evaluated at earlier stages of drug product development.

## 6. Novel Technologies

Recent advancements have introduced innovative techniques for enhancing dry powder inhalers (DPIs), promising improved particle properties and therapeutic ingredient delivery (Table 2). One such method is Particle Replication in Non-Wetting Templates (PRINT), utilizing soft lithography. Employing perfluoropolyether elastomers as molding templates on a silicone master plate, PRINT generates micro- to nano-sized particulate matter with diverse shapes. Researchers have applied this technology to prepare zanamivir-loaded microparticles, resulting in significantly enhanced flow properties compared with conventional DPIs.

- Particle Replication in Non-Wetting Templates (PRINT): PRINT technology represents a cutting-edge approach to improving particle properties by leveraging the principles of soft lithography and allows for precise control over particle size, shape, and surface characteristics. The process involves using perfluoropolyether elastomers as molding templates on a silicone master plate, where preparticle material is filled into molds and pulled out from them using an adhesive [144]. PRINT technology helps in creating micro- to nano-sized particulate matter with desired properties and most importantly with uniform PSD. Additionally, other particle properties such as surface roughness and porosity can also be controlled, leading to improved powder flowability and dispersibility resulting in enhanced DPI performance.
- Thin-Film Freezing (TFF) Technology: The TFF technique stands as a novel advancement in the realm of dry powder inhalation (DPI) formulations. This method involves the fast freezing of a liquid formulation containing the active pharmaceutical ingredient (API) and stabilizer solution within a controlled fluid dynamic system. Upon application, the liquid formulation is quickly spread as a thin film onto a cryogenically frozen surface, inducing the rapid conversion of liquid droplets into solid frozen droplets of 2–3 mm in diameter [145]. The frozen mass is then lyophilized, resulting in dry powder particles with a particle size as small as 200 nm, suitable for inhalation [146]. Moreover, TFF allows the incorporation of higher drug loads into the formulation, which is difficult to achieve by other conventional methods and results in homogenous batches with lower variation in particle properties [147]. TFF technology could be highly beneficial for formulations requiring high drug load, small particle size, and complete amorphization. In a study by Praphawatvet et al. (2022), it was demonstrated that voriconazole particles produced using TFF were low-density and composed of nanoaggregates with improved FPF (73.6 ± 3.2%1) and MMAD (3.03 ± 0.17 μm) [145]. Similarly, other studies also depicted improved inhalation performance using TFF technology [47,148,149]. However, TFF technology requires the preparation of a solution containing API and other excipients in an organic solvent, such as ACN, which could be challenging for some of the manufacturing processes. Additionally, in some cases, due to the amorphous nature of the produced material, it could lead to a decrease in stability by moisture sorption. However, this could be handled by using excipients, such as mannitol or trehalose instead of lactose [150]. The TFF process is required to be optimized for each compound due to changes in solubility in different solvents, which consequently changes the fluid dynamics of the solvent–API system [151].
- Ink JET Printing (IJP): IJP has emerged as a promising technique for the formulation of various types of dosage forms, such as tablets and size-controlled particles [152,153]. IJP becomes really useful in controlling particle morphology in the development of inhalation particles. In this method, liquid formulations containing the API and excipients are deposited dropwise onto suitable substrates using a digital imaging system. By precisely controlling the deposition pattern and composition of the liquid droplets, IJP allows for the creation of particles with defined sizes, shapes, and surface properties. One notable example of the application of IJP in inhalation particle preparation is the development of salbutamol sulfate-loaded alginate aerogel microspheres. Researchers utilized IJP to deposit liquid materials containing salbutamol sulfate onto substrates, resulting in the formation of spherical microspheres with high porosity. These aerogel microspheres exhibited enhanced fine particle fraction (FPF) compared with powders produced using conventional methods, highlighting the effectiveness of IJP in tailoring particle morphology for improved aerosolization efficiency and pulmonary delivery. In conclusion, particle engineering plays a pivotal role in optimizing DPI formulations, ensuring effective drug delivery and therapeutic efficacy. Continued research and innovation in this field are essential for addressing current challenges and advancing the development of next-generation DPIs tailored to meet the evolving needs of patients with respiratory conditions.

**Table 2:** DPI performance optimization techniques evaluated during different studies.

Study Reference	Optimization Mechanism	Formulation Components/Product
Steckel, et al., 1997 [26]	Modification of carrier lactose size	Spinhaler and Easyhaler
Kaialy et al., 2014 [36]	Modification of carrier physical properties by spray-drying	Spray-dried mannitol and albuterol sulfate
Kaialy et al., 2011 [44]	Modification of particle shape	Mannitol and salbutamol sulfate
Zeng et al., 2000 [45]	Modification of carrier and API shape	Lactose and salbutamol sulfate
Singh et al., 2015 [54]	Addition of force control agents	Surface-modified lactose (SML) and fluticasone propionate
Spahn et al., 2022 [63]	Blending equipment	Rifampicin, Magnesium stearate and lactose
Hertel et al., 2017 [94]	Blending parameters	Inhalac 70, Inhalac 400, and Inhalac 230
Steckel et al., 2006 [107]	Milling conditions	Lactose crystals
Brodka-Pfeiffer et al., 2003 [112]	Ball milling parameters	Salbutamol sulphate
Sibum et al., 2020 [122]	Filling process optimization using Omnidose	Amikacin formulation
Stranzinger et al., 2023 [123]	Filling process optimization	Lactohale 300, Lactohale 220 and Inhalac 500
Rabban and Seville 2005 [135]	Spray-drying	β-estradiol, Leucine
Lee et al., 2016 [136]	Spray-drying and jet milling	Bosentan hydrate
Richardson et al., 2007 [141]	Supercritical Fluid Processing (SCF)	Salbutamol sulfate
Rehman et al., 2004 [142]	Supercritical Fluid Processing (SCF)	Terbutaline sulfate
Praphawatvet et al., 2022 [145]	Thin-Film Freezing (TFF) technology	Voriconazole
Mangal et al., 2019 [109]	Co-jet milling	Ciprofloxacin HCl, magnesium stearate, Leucine
Ljsebaert et al., 2001 [143]	Electrohydrodynamic Atomization	Beclomethasone dipropionate

## 7. Conclusions

This article attempts to present a detailed review of the optimization of DPI performance by evaluating various factors, such as formulation optimization, device considerations, and optimization of process parameters. DPIs have demonstrated success in pulmonary drug delivery for treating local lung diseases like COPD, asthma, and cystic fibrosis, or for systemic responses, such as Parkinson’s disease or diabetes mellitus. For an effective drug delivery to the lung, the API particles should be small enough to not settle in the mouth–throat region, and this small size of the API results in poor powder flow properties. Therefore, adding a coarse carrier helps in improving the flow properties and fluidization characteristics of the formulation. A careful selection of formulation components, such as the type of carrier lactose (milled, sieved, or spray dried), additional components (FCAs, fines), or the API to improve the aerosol performance is recommended. In addition to this, the inhaler device forms an essential component of the DPI formulation and significantly affects overall performance. Different device designs should be evaluated to identify the device design with optimum performance, and computational modeling and simulation could be useful tools to facilitate the device selection process. Computational modeling tools such as CFD and DEM modeling have been used extensively and have delivered promising results. Moreover, it is important to consider the influence of downstream processing on DPI performance, as DPI manufacturing relies heavily on unit operations such as micronization, blending, and encapsulation or capsule filling, which makes it critical to evaluate the influence of process parameters on DPI performance. There are multiple particle engineering and novel manufacturing techniques that could be useful in significantly improving DPI performance. In conclusion, DPI formulation performance can be improved by optimizing formulation components and process parameters and utilizing different particle engineering techniques.

## Figures and Tables

**Table 1:** Most commonly used excipients for DPI formulation development.

Excipients	Functionality	Development Stage
Lactose Monohydrate [18]	Carrier, enhances flowability and dispersion	Marketed (Pulmicort Flexhaler, Spiriva Handihaler, Advair Diskus)
Mannitol [18]	Carrier and an alternative to lactose	Marketed (e.g., Exubera)
Glucose [20,21,23]	Carrier	Marketed (e.g., BronchoDual)
Trehalose [20,21,23]	Bulking agent for low-dose APIs, Stabilize sensitive drugs (proteins, peptides)	Underdevelopment
Leucine, Trileucine, Isoleucine [62]	Stabilizer and enhances aerosolization (forms hydrophobic shell during spray drying)	Underdevelopment
Magnesium Stearate [63]	Carrier coating, reduces cohesive forces	Marketed (e.g., SkyeProtect™)
Poloxamer [64]	As a surfactant for the formation of light and porous particles	Underdevelopment
Bile Salts [65]		

## Abbreviations

∆P	Pressure drop across the inhaler
%RSD	Relative Standard Deviation
API	Active Pharmaceutical Ingredient
BAM	Breath-Actuated Mechanism
BSA	Bovine Serum Albumin
CAB	Cohesive Adhesive Balance
CFD	Computational Fluid Dynamics
Ds	Surface fractal dimension
DD	Delivered Dose
DPI	Dry Powder Inhaler
ED	Emitted dose
Edispersion	Energy of Dispersion
ER	Elongation ratio
FCA	Force Control Agent
FPD	Fine Particle Dose
FPF	Fine Particle Fraction
GRAS	Generally Recognized as Safe
HPMC	Hydroxypropyl methylcellulose
L/min	Liter per minute
MgSt	Magnesium stearate
NGI	Next-Generation Impactor
pMDIs	Pressurized Meter Dose Inhaler
ToF-SIMS	Time-of-flight secondary ion mass spectrometry
t_dispersion_	Time for Dispersion
V_af_	Volume airflow
XPS	X-ray photoelectron spectroscopy
